# Effects of Commercially Available Dietary Supplements on Resting Energy Expenditure: A Brief Report

**DOI:** 10.1155/2014/650264

**Published:** 2014-01-02

**Authors:** Roger A. Vaughan, Carole A. Conn, Christine M. Mermier

**Affiliations:** ^1^Department of Health, Exercise and Sports Science, University of New Mexico, USA; ^2^Department of Biochemistry and Molecular Biology, University of New Mexico Health Sciences Center, USA; ^3^Department of IFCE: Nutrition, University of New Mexico, USA

## Abstract

Commercially available dietary products advertised to promote weight loss are an underresearched but heavily purchased commodity in the United States. Despite only limited evidence, interest in dietary supplements continues to increase. This work uniquely summarizes the current evidence evaluating the efficacy of several over-the-counter thermogenic products for their effects on resting energy expenditure. Currently, there is some evidence suggesting dietary products containing select ingredients can increase energy expenditure in healthy young people immediately following consumption (within 6 hours). It is unclear if supplement-induced increases in metabolic rate provide additional benefit beyond that provided by dietary constituents that contain similar ingredients. It is also unclear if dietary supplements are effective for weight loss in humans.

## 1. Introduction

Obesity has rapidly become a leading cause of death without a foreseeable resolution in the near future. Most clinicians believe the combination of food overconsumption with sedentary lifestyle synergistically promote weight gain and obesity. The importance of restrictive dietary practices in combination with physical activity are of undeniable importance for weight loss and general health [1, 2]; however, the role of genomics and corresponding interactions with dietary and exercise practice still remain largely ill-defined as the field is still in its infancy [3]. A variety of therapies are currently available to combat obesity; however recent trends in obesity prevalence provide strong evidence that current interventions are insufficient to effectively slow the development of obesity and related comorbidities [1–3].

The role which food chemicals and dietary components may play in obesity therapeutics is increasing interest. In obesity research, several classes of chemicals including methylxanthines, polyphenols, capsaicin (capsaicinoids/capsinoids), polyunsaturated fats, and many other components found in food have shown some promise in promoting a metabolic advantage for weight loss [4, 5]. As a result of preliminary data supporting some of the aforementioned ingredients, producers of commercially available dietary supplements often include one or many of these components in their products [5]. Dietary supplements are sold for a variety of purposes, including weight loss, and many ingredients are promoted specific benefits such as increased thermogenesis.

Dietary supplements are unique because unlike pharmaceutical agents, they do not require close regulation of content, function, or safety prior to consumption by humans [6] and are generally not recommended by healthcare professionals [7]. Because of limited regulation, it is not uncommon for single ingredients to be promoted for a promiscuous variety of unsubstantiated functions and health benefits. Although subjected to limited regulation, it is mandated that all dietary supplements be marked with a disclaimer stating the lack of support by the FDA for all claims. Supplements sold as thermogenic products for weight loss and/or energy augmenters are often promoted to cause rapid weight loss, often times independent of calorie restriction or physical activity. Despite resistance from healthcare professionals, consumer interest in dietary supplements continues to surge [8]. According to NHANES questionnaire data from roughly 12,000 participants, approximately 50% of those surveyed used dietary supplements in the past 30 days [8]. Use was most common among 20–30-year-old participants, and more common in women than in men [8]. Interestingly when asked to select the motivating factor for supplement consumption, “weight loss” or “get more energy” were reported as the motivation for approximately 14% of participants, both of which were also more common in women participants [8]. This work seeks to summarize current research evaluating commercially available dietary supplements sold as stimulators of thermogenesis and increased metabolic rate leading to weight loss.

## 2. Individual Ingredients as Metabolic Stimulators

### 2.1. Caffeine and Xanthine Metabolites

Of the available dietary supplements marketed for weight loss, many contain a blend of ingredients that includes caffeine. Caffeine has previously been shown to dose-dependently heighten resting energy expenditure in adult humans, both normal and overweight [5, 9–14]. Despite some conflicting data, it is generally accepted that caffeine effectively stimulates the central nervous system and increases metabolic rate in humans [14–16]. Caffeine functions through inhibition of phosphodiesterase (PDE) and through stimulation of adenosine receptors, leading to accumulation of intracellular 3,5-cyclic-adenosine monophosphate (cAMP) which is metabolically excitatory for cells [11, 12, 14].

### 2.2. *β*_**2**_ -Adrenergic Receptor Agonists

Like caffeine, stimulators of *β*_2_-adrenergic receptors such as ephedra/ephedrine have been another primary component found in supplemental thermogenic products [9, 11, 14]. Following the restriction of ephedra use, similar chemical analogs such as synephrine from bitter orange extract became widely accepted replacements [17–20]. *β*_2_-adrenergic receptor agonists work to increase cAMP biosynthesis, while caffeine inhibits the breakdown of cAMP by PDE [14]. Additionally, salicylates inhibit prostaglandins further allowing the accumulation of cAMP [14]. Taken together, caffeine, *β*_2_-adrenergic receptor agonists and salicylates function to synergistically increase cAMP accumulation and metabolic rate [14].

### 2.3. Green Tea Extract Polyphenols

Several other dietary stimulators of metabolism are thought to function by augmenting the accumulation of cAMP [14]. Green tea extract is rich in polyphenols and is heavily promoted for health benefits including increased metabolism [4, 12–14, 21–28]. Epigallocatechin gallate (EGCG), epigallocatechin (EGC), epicatechin gallate (ECG), and epicatechin (EC) are believed to cause most of green tea's beneficial effects [5]. Other teas such as black tea, thearubigins, and theaflavins share similar effects to those found in green tea [4, 29]. Green tea extract appears to offer some thermogenic effect when coupled with caffeine resulting in increased energy expenditure [14, 22, 30]. In contrast, isolated green tea polyphenol ingestion independent of caffeine does not appear to increase metabolic rate; however it does increase indicators of fat metabolism in some populations [14, 22, 30].

Green tea is purported to function by inhibiting the degradation of *β*_2_-adrenergic receptor agonists such as norepinephrine by the enzyme catechol O-methyltransferase (COMT), thereby increasing intracellular cAMP [4, 12]. Again, the combination of green tea polyphenols with caffeine cause a synergistic effect further increasing cAMP. Recently, the effects and mechanisms of green tea extract in human metabolism were reviewed and it was suggested that the mechanism of COMT inhibition is possibly a product of *in vitro* experimental conditions, because COMT inhibition experiments have yet to identify a specific catechin inhibitor, or determine if the active polyphenol is an inhibitor, a substrate of COMT, or a combination [31]. Additionally, the mechanisms of the hypothesized downstream effect of COMT inhibition resulting in heightened fat metabolism have yet to be elucidated experimentally [31]. In addition to COMT inhibition, it is theorized that effects of green tea extract are also a function of metabolic gene activation. Specifically, green tea may function to inhibit adipogenic genes such as peroxisome proliferator-activated receptor (PPAR*γ*) and sterol regulatory element binding protein-1c (SREBP-1c) and increase the expression of genes that increase energy expenditure and mitochondrial biogenesis including nuclear respiratory factors (NRF), mitochondrial uncoupling protein-3 (UCP3), peroxisome proliferator-activated receptor (PPAR*α*), and peroxisome proliferator-activated receptor *γ* coactivator-1*α* (PGC-1*α*), leading to increased oxidative and metabolic capacities [31].

### 2.4. Capsaicinoids and Capsinoids

**Figure 1:**
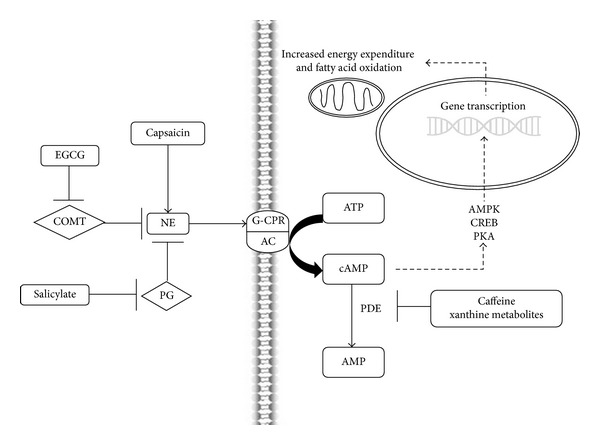
Summary of proposed mechanisms of common ingredients in thermogenic products. adenylate cyclase (AC), adenosine monophosphate (AMP), 3,5-cyclic-adenosine monophosphate (cAMP), 5′adenosine monophosphate-activated protein kinase (AMPK), adenosine triphosphate (ATP), catechol O-methyltransferase (COMT), cAMP-related element binding protein (CREB), epigallocatechin gallate (EGCG), G-coupled protein receptor (G-CPR), prostaglandin (PG), protein kinase A (PKA), norepinephrine (NE), and phosphodiesterase (PDE).

Another common constituent of thermogenic products is the capsaicinoids/capsinoids, components found in spicy foods such as chili pepper and cayenne [32, 33]. Available data suggests that supplemental capsaicinoids/capsinoids effectively increase resting energy expenditure, although findings have been inconsistent [32–34]. Capsaicinoids are believed to function by increasing catecholamine release, heightening sensitivity to circulating catecholamines, or a combination thereof [33].  Figure 1 summarizes the proposed mechanisms of the abovementioned individual ingredients commonly found in commercially available thermogenic products.

## 3. Propriety and Commercially Available Thermogenic Products

### 3.1. Proprietary Products

Several previous investigations have identified that commercially available products increase resting energy expenditure in a variety of subjects, most frequently using young healthy subjects (Table 1). Most commonly these products contain several of the following: caffeine, green tea, capsaicin, and catecholamine-like compounds (synephrine). In general, most investigations evaluated changes in energy expenditure within 6 hours of consumption. While the magnitude of the individual supplement's effect on energy expenditure seems to vary depending on the ingredients consumed, the increase in energy expenditure is similar to previous observations using individual ingredients such as caffeine, ranging from 50 to 200 k cals per hour immediately following ingestion. Although this increase is experimentally significant, it must be stressed that the effect is time-dependent and energy expenditure appears to return to basal levels 6 hours following consumption. Previous acute (24 hours or less) investigations with caffeine alone or green tea with caffeine showed approximately a 4% increase in 24 hour metabolic rate [5, 10, 12, 14] which approximates the increases seen with selected thermogenic products. Extended treatment for up to 8 weeks has also shown increased resting energy expenditure, suggesting that regular green tea consumption with select stimulants can continuously cause an increase in metabolism [35, 36].

Despite evidence that several of the single ingredients previously described have been shown to increase metabolic rate, there is some question about the efficacy of advertised dietary products for weight reduction. Hasani-Ranjbar and colleagues selectively identified dozens of studies which implicate natural products in the reduction of body weight in humans [6]. The investigations reviewed ingredients frequently found in dietary supplements including caffeine, ephedra, and a variety of herbal agents, many of which function in part as antioxidants [6]. These studies included experimental designs lasting from as little as 4 weeks to up to 9 months without controlling for exercise and/or concurrent energy restriction. In general, there appears to be some evidence that thermogenic products may partially aid in weight loss, although it is difficult to identify the magnitude because of between-study variables.

## 4. Limitations to Current Observations

### 4.1. Individual Ingredients

One possible contributor to discrepancies within the literature regarding efficacy of individual ingredients is experimental variability. Multiple confounds exist in energy expenditure measurements such as duration of measurement, type of analytical and measurement software, duration of measurement after ingestion of stimulant, and the amount of purported metabolic stimulator consumed. An additional issue is that much of the original measurements were conducted several decades ago. Moreover, significant biological differences between demographics may also contribute to varied response across similar studies.

### 4.2. Commercial Products

Proprietary blends found within dietary supplements have numerous issues that are not encountered when testing pharmaceutical grade single chemicals (such as caffeine). For example, dietary supplements may vary in content from one lot to the next, suggesting some batches function better than others. Additionally, supplements are not required to third-party test products for purity and content, which allows for the possibility that nonlisted ingredients are included and ingredients that are purportedly included are missing. Such inconsistencies would likely inflate the variability of experimental measurement, contributing to inconsistencies in the reported effects of dietary supplements. Perhaps the most significant limitation to commercially available dietary supplements is the near-complete absence of scholarly evidence supporting individual product safety and efficacy. Another interesting and significant variable worth discussing is the matter of funding for research on dietary supplements. Multiple articles disclose that funding is contributed by the companies which market the product for retail purposes. This is not surprising because cost of biological research continues to increase while funding opportunities remain limited; therefore it is logical that select companies have outsourced primary research opportunities to the university setting. Surprisingly, many dietary supplements advertise one or multiple nonbiased studies illustrating efficacy of their product; however often times these studies have not been systematically peer-reviewed or readily shared with the scientific community making the original research difficult to locate. In addition, identification and tracking of adverse events with the majority of studies would not meet requirements for similar pharmaceutical agents [6]. Of the known adverse effects, dry mouth, insomnia, nervousness, palpitations, headache hypertension, arrhythmias, myocardial infarction, stroke, and seizures have been linked to consumption of ingredients such as caffeine and ephedra, suggesting a major need for further safety evaluation prior to consumption [6, 7]. Lastly, along with limited evidence of whole-body efficacy and safety, there is inadequate evaluation of the molecular effects of commercially available dietary supplements. Our lab previously demonstrated that select dietary supplements containing a variety of ingredients could effectively increase skeletal muscle metabolism and mitochondrial content *in vitro*; however additional information is required to adequately describe cell- and tissue-specific effects of thermogenic products [45, 46].

## 5. Concluding Remarks

Increasing obesity prevalence and consumer interest in thermogenic dietary supplements support the need for further research of these and other supplement ingredients. From the available evidence, it appears that commercially available dietary supplements advertised to stimulate metabolism have the propensity to increase metabolic rate. Despite significant increases in resting energy expenditure, it is doubtful that commercially available thermogenic products stimulate metabolism more than consumption of food products containing equivocal content of caffeine/stimulants and/or polyphenols. Moreover, it should be mentioned that increases in metabolism induced by food or dietary supplement are small, contributing only subtly to metabolic rate. Additional research is necessary to identify the precise mechanisms by which commonly consumed ingredients function to alter energy expenditure and what corresponding molecular adaptations may develop.

## Figures and Tables

**Table 1:** Investigations evaluating the effects of thermogenic products on resting energy expenditure (REE) following acute (24 hours or less) and extended (longer than 24 hours).

Subjects	Ingredients	Findings	Author
Effects of acute consumption of dietary supplements on energy expenditure			

Obese men (*N* = 19)	Green tea extract 750 mg L-tyrosine 609 mg caffeine 151 mg cayenne 225 mg calcium 1965 mg	Supplement containing bioactive food ingredients increased daily REE by 200 kJ or 2% (48 kcal)	Belza and Jessen 2005 [34]

Healthy men and women (*N* = 20)	Caffeine anhydrous toothed clubmoss (*Huperzia serrata*) yerba mate (*Llex paraguariensis*) 3′-5′-CAMP (3′-5′-cyclic adenosine monophosphate) synephrine HCL R-beta-methylphenylethylamine N-Methyl-B-phenylethylamine yohimbe Methyl-hordenine	Increased REE 59 ± 26 kcal·6 hrs, serum epinephrine, norepinephrine, glycerol, systolic and diastolic BP	Bloomer et al. 2009 [37]

Healthy men and women (*N* = 10)	Caffeine anhydrous toothed clubmoss (*Huperzia serrata*) yerba mate (*Llex Paraguariensis*) 3′-5′-CAMP (3′-5′-cyclic adenosine monophosphate) synephrine HCL R-beta-methylphenylethylamine N-mehtyl-B-phenylethylamine yohimbe Methyl-hordenine	Increased REE, increased HR, no change in BP	Hoffman et al. 2009 [38]

Healthy men and women (*N* = 12)	Caffeine anhydrous toothed clubmoss (*Huperzia serrata*) yerba mate (*Llex paraguariensis*) 3′-5′-CAMP (3′-5′-cyclic adenosine monophosphate) synephrine HCL R-beta-methylphenylethylamine N-mehtyl-B-phenylethylamine yohimbe Methyl-hordenine	Increased REE 45, 60, 120 minutes after ingestion with no change in HR or BP	Jitomir et al. 2008 [39]

Healthy men and women (*N* = 8)	Caffeine anhydrous, guarana, yerba mate green tea extract, L-carnitine L-tartrate, pathothenic acid, chromium picolinate, proprietary blends containins, AssuriTea green tea extract, *Salvia sclarea*, raspberry ketones and *Capsicum annum* extract, plus l-tyrosine, *Salix alba*, *Zingiber officinale*, *fucus vesiculosus*, *Panax ginseng*, bioperineW	Increased REE 60, 120, 180, 240 minutes after ingestion	Outlaw et al. 2013 [40]

Healthy men and women (*N* = 28)	Supplement caffeine 200 mg, capsicum extract 33.34 mg, Niacin 20 mg, Bioperine 5 mg	Increased REE 50 minutes after consumption with elevated HR and BP	Ryan et al. 2009 [32]

40 male and 40 female healthy young subjects	1 mg capsinoids in 199 mg of rapeseed oil and medium-chain triglycerides	No change in REE or weight but increased fat oxidation	Snitker et al. 2009 [41]

10 healthy subjects per treatment	600 mg naringin, 50 mg *p*-synephrine 100 mg hesperidin, 50 mg *p*-synephrine, 600 mg naringin 1000 mg hesperidin, 50 mg *p*-synephrine, 600 mg naringin	Increased REE 129 kcal Increased REE 183 kcal Increased REE 79 kcal	Stohs et al. 2011 [17]

8 male and 10 female healthy young subjects	442 mg of a proprietary: 100 mg of caffeine, 230 mg of green tea extract, L-tyrosine, L-taurine, chocamine, white willow extract, yohimbine-HCl, vinpocetine	Increased REE 60, 120, 180 minutes after ingestion	Wilborn et al. 2009 [42]

Healthy men and women (*N* = 60)	Caffeine, citrus aurantium, garcinia, cambogia and chromium polynicotinate	Significantly increased REE at 60, 120, 180 min	Dalbo et al. 2008 [43]

18 healthy young men	1.5 mg capsinoids in 199 mg of rapeseed oil and medium-chain triglycerides	EE increased by 15.2 kJ/h (BAT+) (5 kcals/hour) groups 1 hours after ingestion	Yoneshiro et al. 2012 [44]

Effects of extended consumption of dietary supplements on energy expenditure			

Healthy men and women (*N* = 8)	pantothenic acid, 40 mg; green tea leaf extract 200 mg; guarana extract (198 mg of caffeine), 550 mg; bitter orange (9 mg of synephrine), 150 mg; white willow bark extract (7.5 mg of salicin), 50 mg; ginger root, 10 mg; proprietary blend (L-tyrosine, L-carnitine, naringin), 375 mg, phenylephrine (20 mg)	Increased REE with increased body weight	Greenway et al. 2006 [36]

Obese subjects (*N* = 80)	Green tea extract 750 mg L-tyrosine 609 mg caffeine 151 mg Cayenne 225 mg calcium 1965 mg	Supplement increased REE by 87.3 kJ (21 kcal) sustained for 8 weeks with reduced body fat mass	Belza et al. 2007 [35]

## Abbreviations

AC: Adenylate cyclase
AMP: Adenosine monophosphate
AMPK: 5′Adenosine monophosphate-activated protein kinase
ATP: Adenosine triphosphate
cAMP: 3,5-cyclic-adenosine monophosphate
COMT: Catechol O-methyltransferase
CREB: cAMP-related element binding protein
EGCG: Epigallocatechin gallate
EGC: Epigallocatechin
ECG: Epicatechin gallate
EC: Epicatechin
G-CPR: G-Coupled protein recepetor
PG: Prostaglandin
PKA: Protein kinase A
NE: Norepinephrine
NRF: Nuclear respiratory factors
PGC-1*α*: Peroxisome proliferator-activated receptor *γ* coactivator-1*α*
PPAR*α*: Peroxisome proliferator-activated receptor *α*
PPAR*γ*: Peroxisome proliferator-activated receptor *γ*
PDE: Phosphodiesterase
SREBP-1c: Sterol regulatory element binding protein-1c
UCP3: Mitochondrial uncoupling protein-3.
